# Long-Term Velopharyngeal Insufficiency–Related Quality of Life in Cleft Palate Patients: Speech and Surgical Factors

**DOI:** 10.1016/j.identj.2025.109354

**Published:** 2026-01-10

**Authors:** Xiaobao Dang, Hongzheng Gu, Xin Zhao, Ruiqing Jiang, Hanyao Huang, Karim Ahmed Sakran

**Affiliations:** a Department of Oral and Maxillofacial Surgery, Zhenjiang Stomatological Hospital, Zhenjiang, Jiangsu, China; b Department of Oral and Maxillofacial Surgery, West China Hospital of Stomatology, Sichuan University, Chengdu, Sichuan, China

**Keywords:** Cleft palate, Fistula, Quality of life, Surgical repair, Velopharyngeal insufficiency

## Abstract

**Introduction and aims:**

Velopharyngeal insufficiency (VPI) is a common functional complication in patients with cleft palate, often resulting in impaired speech, social difficulty, and psychosocial distress. This study aims to assess VPI–related quality of life (VPI-QOL) in cleft palate patients after primary palatoplasty, and to identify clinical and functional predictors, particularly speech outcomes and postoperative complications, that significantly affect the perceived QOL.

**Methods:**

This cross-sectional study involved 170 cleft palate patients assessed using the validated Chinese version of the VPI Effects on Life Outcomes (VELO) instrument. Both parent-proxy and youth self-reports were collected. Patients were stratified by QOL status into adequate and inadequate QOL groups. Demographic, surgical, and anatomical variables, along with professional speech evaluations, were analysed. Statistical comparisons used Chi-square and Mann–Whitney *U* tests, with multivariate logistic regression to identify independent predictors of poor QOL.

**Results:**

Among 170 patients, 107 (62.9%) had adequate and 63 (37.1%) had inadequate QOL. Inadequate QOL was associated with older age at surgery (mean 5.19 vs 1.98 years; *P* < .001) and at evaluation (*P* < .01). Oronasal fistula (ONF) occurred in 14.7%, and VPI in 30%. Delayed palatoplasty, presence of ONF, and poor speech intelligibility were significantly linked to lower VELO scores. ONF (odds ratio = 8.531, *P* < .001) and speech intelligibility deficits (odds ratio = 8.854, *P* < .001) were independent predictors of poor QOL. Patients with VPI reported significantly lower scores across all VELO domains. Youth and parental responses were generally consistent, with parents noting more pronounced emotional and social effects.

**Conclusion:**

Speech deficits and postoperative complications, particularly ONF and reduced intelligibility, are major determinants of VPI-related QOL. Timely surgery, prevention of ONF, and targeted speech intervention are essential for optimizing both functional and psychosocial outcomes.

**Clinical relevance:**

Speech intelligibility and ONF strongly influence VPI-QOL; early surgery, ONF management, speech therapy, and VELO use improve outcomes and guide multidisciplinary cleft care.

## Introduction

Cleft lip and palate represent some of the most prevalent congenital anomalies affecting the craniofacial region.^1^ Management typically requires a multidisciplinary approach due to the broad spectrum of associated challenges, including speech and language impairments, craniofacial abnormalities, dental anomalies, malocclusion, hearing issues, developmental delays, and psychosocial difficulties. Velopharyngeal insufficiency (VPI) is a common functional complication following primary palatoplasty in patients with cleft palate, often resulting in impaired speech, nasal air emission, swallowing difficulty, and psychosocial distress.^2^^,^^3^ VPI stems from incomplete closure between the soft palate and pharyngeal wall during speech or swallowing and is particularly prevalent among individuals with cleft palate due to structural anomalies of the velopharyngeal mechanism.^3^, ^4^, ^5^ Despite successful anatomical repair, up to 20% to 30% of patients with repaired cleft palate continue to experience residual VPI, indicating the need for ongoing assessment and intervention.^6^

Health-related quality of life (QOL) has emerged as a key outcome in cleft care, reflecting a patient’s perception of the impact of their condition on functional, emotional, and social well-being.^7^^,^^8^ Traditional measures of surgical success, such as anatomical closure, speech articulation scores, and perceptual speech assessments, provide valuable objective data but may fail to fully capture the patient’s lived experience, particularly in children.^9^^,^^10^ Increasing attention is therefore being directed towards patient-reported outcome measures (PROMs), which serve as essential tools for evaluating long-term rehabilitation and guiding personalized care planning.^11^, ^12^, ^13^

Among the available instruments, the VPI Effects on Life Outcomes (VELO) questionnaire, developed by Skirko et al,^4^^,^^14^, ^15^, ^16^ has proven to be a reliable, valid, and sensitive tool for assessing VPI-related QOL. VELO is a disease-specific PROM tailored for paediatric populations with VPI and offers both parent-proxy and youth-report formats. It covers multiple life domains, including speech, swallowing, emotional impact, situational difficulty, social perception, and caregiver burden. Compared to broader health-related QOL instruments such as the COHIP or COHQOL, VELO provides more targeted insight into the VPI-specific challenges experienced by patients and families.^17^

Recent efforts have focused on adapting VELO into multiple languages, including Mandarin Chinese, to improve accessibility and global utility.^18^^,^^19^ Validation studies of the Chinese version have shown excellent internal consistency, discriminant validity, and responsiveness across multiple domains. Moreover, previous studies confirmed significant inverse associations between VELO scores and speech parameters such as intelligibility, hypernasality, and nasal air emission, supporting both the instrument’s criterion validity and its clinical relevance in guiding postoperative care.^15^^,^^18^^,^^20^ In addition, researchers have emphasized the value of establishing VELO score cutoffs to identify patients in need of further intervention, suggesting its potential role in clinical decision-making.^4^

Despite these advances, few studies have examined how specific clinical factors, such as surgical timing, oronasal fistula (ONF), and professional speech assessment, correlate with VELO-based QOL outcomes in cleft palate patients postprimary palatoplasty. ONF remains a persistent postoperative complication that not only compromises velopharyngeal function but also leads to nasal regurgitation, speech distortion, and social embarrassment.^1^^,^^21^ Likewise, speech intelligibility has consistently been identified as one of the strongest predictors of perceived QOL among children with cleft-related VPI.^14^^,^^20^

Therefore, this study aims to evaluate the VPI-related QOL in cleft palate patients after primary palatoplasty using the VELO instrument and to identify key predictors of inadequate QOL, with a particular focus on speech outcomes and surgical complications.

## Methods

### Study design and participants

This cross-sectional study was conducted on patients with cleft palate following institutional ethical approval (No. WCHSIRB-D-2022-409) and adhered to the principles of the Declaration of Helsinki. Written consent was obtained from all participants. A total of 170 nonsyndromic cleft palate patients who underwent primary palatoplasty between 2011 and 2017 were retrospectively reviewed and included. Eligible patients, across all age groups, met the following criteria: (1) nonsyndromic, isolated or combined cleft palate patients (including soft cleft palate, hard and soft cleft palate, unilateral cleft lip and palate, and bilateral cleft lip and palate); and (2) availability of complete clinical, surgical, and follow-up data. Exclusion criteria included submucous cleft palate, secondary palate repair, significant hearing impairment, or cognitive delay interfering with participation in quality-of-life assessments. All patients were treated at a single high-volume tertiary cleft centre. All surgeries were performed by two experienced cleft surgeons from the same multidisciplinary team using a standardized palatoplasty technique incorporating radical palatal muscle reconstruction, soft palate lengthening, and mucoperiosteal flap mobilization, following the principles of the Furlow and intravelar veloplasty methods as previously described by Sakran et al.^22^ It should be noted that none of the included patients received standardized postoperative speech therapy as part of their management.

### Speech and functional assessments

Speech outcomes were evaluated after the age of three by two certified speech-language pathologists (SLPs), using both perceptual assessment and nasopharyngoscopy, following previously described standardized protocols.^23^^,^^24^ Speech intelligibility deficit, hypernasality, and nasal emission were assessed perceptually through the whole spontaneous and conversational speech sample by estimating the hoarseness and volume of voice, speech tone, and articulation. Velopharyngeal function was estimated based on resonance severity and nasal air emission as well as nasal endoscopy. All speech parameters were rated using a four-point scale: 0, none, normal, or within acceptable limits; 1, mild; 2, moderate; or 3, severe, except nasal emission, which was rated using a dichotomous grade (0 = absent, 1 = present). The presence of persistent ONF was diagnosed when a postoperative fistula persisted beyond 6 months and was verified by a senior specialist and two SLPs.

### QOL assessment

VPI-related QOL was measured using the validated Chinese version of the VELO instrument.^18^^,^^19^ The VELO instrument includes two versions, the parent-proxy version (26 items) was completed by caregivers, and the youth version (23 items) by patients aged 8 years and older at the time of evaluation. The VELO instrument encompasses five main domains: speech limitation, swallowing problems, situational difficulty, emotional impact, and perception by others. Responses were recorded using a 5-point Likert scale from 0 (never) to 4 (almost always), and scores were transformed to a 0 to 100 scale, with higher scores indicating better QOL. VELO total scores ≥79.04 in parent and ≥85.77 in youth versions were classified as adequate QOL, while scores <79.04 in parent and <85.77 in youth versions indicated inadequate QOL based on established cutoff values derived from receiver operating characteristic analyses.^18^

### Data collection

Demographic and clinical data collected included patient sex, age at surgery and evaluation, cleft type, cleft width, palate width, soft palate length, pharyngeal cavity depth, operation length, presence of ONF, speech performance scores, as well as VELO scores.

### Statistical analysis

All statistical analyses were performed using SPSS version 25.0 (IBM Corp.). Descriptive statistics were expressed as means ± standard deviations. The Mann–Whitney *U* test was used for continuous data comparisons, and the *χ*² test or Fisher’s exact test for categorical variables. Multivariate logistic regression was employed to determine independent predictors of inadequate VPI-QOL. Inter- and intrarater reliability for the perceptual speech parameters was assessed using quadratic weighted kappa coefficients, while VELO intrarater agreement was evaluated using intraclass correlation coefficients. Each SLP independently assessed all participants, and approximately 15% of cases were re-evaluated after 1 week to determine intrarater consistency. The speech assessments demonstrated high reliability, with intrarater *κ* = 0.92 to 0.95 and inter-rater *κ* = 0.89 to 0.93. VELO responses also showed good intrarater reliability (intraclass correlation coefficients = 0.81-0.90). Given the strong agreement between the two SLPs, the assessment records from one randomly selected SLP were used for the final analysis. Statistical significance was set at *P* < .05. Bonferroni correction was also applied (adjusted *P* = .013-.008) to mitigate overtesting in multiple comparisons.

## Results

### Patient characteristics

Table 1 summarizes the demographic and anatomical characteristics of the 170 patients. Overall, 94 (55.3%) were male, and 76 (44.7%) were female. The mean age at surgery was 3.17 ± 4.92 years (range: 0.20-27), and the age at evaluation (ie, age at the study point) was 8.90 ± 5.77 years (range: 3-29). A total of 107 patients (62.94%) were classified as having adequate QOL, while 63 (37.06%) had inadequate QOL. Among the 75 paired parent–youth responses, agreement was high (*r* = 0.77), with comparable total VELO scores (parents: 78.13 ± 21.0; youth: 80.75 ± 17.51). Only 10 cases (7.5%) showed discordant QOL classifications, all of which reflected adequate QOL by parent report and borderline scores by youth report. In cases of discrepancy, the classification consistent with the professional speech evaluation was applied. Inadequate QOL was significantly associated with older age at palatal repair and at the time of evaluation (*P* < .01). No significant differences were observed between QOL groups regarding sex distribution, cleft type, cleft width, palatal dimensions, or soft palate length. Only pharyngeal cavity depth demonstrated a significant anatomical association with lower QOL (*P* < .01).

**Table 1:** Patient demographics and clinical characteristics stratified by VPI-quality of life (170 patients).

Character	Total/overall	Parental VPI-QOL (170 reports)		Youth VPI-QOL (75 reports)	
		Adequate, 107	Inadequate, 63	Adequate 36	Inadequate 39
Gender, *n* (%)					
Male	94 (100)	60 (63.83)	34 (36.17)	22 (50)	22 (50)
Female	76 (100)	47 (61.84)	29 (38.16)	14 (45.16)	17 (54.84)
Age at repair (y)	3.17 ± 4.92	1.98 ± 2.21	5.19 ± 7.15***	3.19 ± 3.70	7.86 ± 8.15***
Age at evaluation (y)	8.90 ± 5.77	7.94 ± 4.77	10.52 ± 6.87**	11.46 ± 4.13	15.59±5.66***
Follow-up time (y)	5.60 ± 3.82	5.89 ± 3.98	5.10 ± 3.52	8.44 ± 4.54	7.57 ± 4.21
Cleft palate type, *n* (%)					
Isolated (SCP/HSCP)	81 (100)	46 (56.80)	35 (43.2)	7 (33.33)	14 (66.67)
Combined (UCLP/BCLP)	89 (100)	61 (68.54)	28 (31.46)	29 (53.70)	25 (46.3)
Cleft width, mm	11.50 ± 3.38	11.32 ± 3.38	11.83 ± 3.41	12.16 ± 1.96	12.83 ± 3.05
Palatal width, mm	34.98 ± 25.52	32.56 ± 4.75	39.38 ± 42.31	34.05 ± 5.61	35.28 ± 5.91
Soft palate length, mm	12.59 ± 2.89	12.41 ± 2.65	12.92 ± 3.28	13.14 ± 3.36	12.64 ± 2.08
Pharyngeal cavity depth, mm	12.43 ± 2.70	11.95 ± 2.20	13.32 ± 3.26**	12.89 ± 3.00	13.50 ± 3.27
Operation length, min	74.54 ± 19.69	73.48 ± 20.85	76.47 ± 17.42	76.07 ± 5.16	77.60 ± 11.38
Total VELO score	79.14 ± 19.23	91.34 ± 6.44	58.42 ± 15.62	94.87 ± 4.34	67.73 ± 14.74

Statistical variances calculated using Chi-squared test for gender and cleft type, while Mann–Whitney *U* test for other variables. Significant differences: **P value <0.01, ***P value <.001.

BCLP, bilateral cleft lip and palate; HSCP, hard and soft cleft palate; SCP, soft cleft palate; UCLP, unilateral cleft lip and palate; VPI-QOL, velopharyngeal insufficiency-related quality of life; gender and cleft type are reported by frequency and rate, while other variables as mean ± standard deviation.

### Postoperative outcomes and VPI-QOL

Professional postoperative assessments demonstrated strong associations between clinical outcomes and VPI-related QOL (Table 2). The presence of an ONF and poorer speech parameters, particularly reduced intelligibility, hypernasality, nasal emission, and impaired velopharyngeal function, were consistently linked with inadequate QOL (*P* < .001).

**Table 2:** Incidence of professional reported postoperative outcomes stratified by VPI-quality of life.

Outcome	Total	VPI-QOL, *n* (%)		*P* value
		Adequate	Inadequate	
Wound healing				
Complete closure	145	99 (68.28)	46 (31.72)	.001
Oronasal fistula	25	8 (32)	17 (68)	
Speech intelligibility				
Normal	109	91 (83.49)	18 (16.51)	<.001
Mild deficiency	1	1 (100)	0 (0)	
Moderate deficiency	26	6 (23.08)	20 (76.92)	
Severe deficiency	34	9 (26.47)	25 (73.53)	
Velopharyngeal function				
Normal	119	94 (79)	25 (21)	<.001
Mild VPI	14	4 (28.57)	10 (71.43)	
Moderate VPI	29	7 (24.14)	22 (75.86)	
Severe VPI	8	2 (25)	6 (75)	
Hypernasality				
None	110	92 (83.64)	18 (16.36)	<.001
Mild	26	6 (23.08)	20 (76.92)	
Moderate	29	7 (24.14)	22 (75.86)	
Severe	5	2 (40)	3 (60)	
Nasal air emission				
Absent	116	94 (81.03)	22 (18.97)	<.001
Present	54	13 (24.07)	41 (75.93)	

VPI-QOL, velopharyngeal insufficiency-related quality of life. *P* values calculated using Chi-squared test at .05 level. Bonferroni correction was also applied at adjusted *P* value = .013.

### VELO score analysis

As shown in Table 3, patients with postoperative VPI reported substantially lower VELO total and domain scores across both parental and youth reports (*P* < .001). The most affected domains included speech limitation, situational difficulty, emotional impact, and perception by others.

**Table 3:** Comparison of VELO scores as per postoperative velopharyngeal function.

VELO score	Overall	Velopharyngeal function		*P* value
		Normal	Velopharyngeal insufficiency	
Parent total score	79.14 ± 19.23	85.45 ± 14.74	64.41 ± 20.50	˂.001
Domain score				
Speech limitations	74.98 ± 20.44	79.98 ± 10.10	63.31 ± 20.96	˂.001
Swallowing problems	87.21 ± 19.74	91.46 ± 15.01	77.29 ± 25.34	˂.001
Situational difficulty	73.44 ± 25.21	81.18 ± 20.58	55.39 ± 25.96	˂.001
Emotional impact	81.21 ± 25.61	89.39 ± 17.23	62.13 ± 31.39	˂.001
Perception by others	82.06 ± 22.75	88.92 ± 16.72	66.05 ± 26.76	˂.001
Caregiver impact	77.70 ± 23.09	82.21 ± 20.55	67.26 ± 25.35	˂.001
Youth total score	80.75 ± 17.52	85.85 ± 15.09	71.70 ± 18.12	˂.001
Domain score				
Speech limitations	75.24 ± 21.56	79.17 ± 21.65	68.25 ± 19.92	.006
Swallowing problems	90.44 ± 13.54	92.36 ± 10.57	87.04 ± 17.35	.148
Situational difficulty	78.80 ± 20.97	84.79 ± 17.80	68.15 ± 22.24	˂.001
Emotional impact	81.25 ± 22.42	88.41 ± 16.61	68.52 ± 25.86	˂.001
Perception by others	85.42 ± 20.20	91.41 ± 15.77	74.77 ± 22.96	˂.001

*P* value calculated using Mann–Whitney *U* Test at significant level .05. Bonferroni correction was also applied at adjusted *P* value = .008.

### Domain item-level scores

Itemized analysis (Table 4) revealed that both parents and children consistently reported lowest scores on items related to speech clarity and intelligibility in daily social situations, such as ‘trouble being understood when in a hurry’ and ‘speech sounding different from other children’. Emotional and social consequences, such as ‘being teased’, ‘getting frustrated’, and ‘being treated as less intelligent’, also scored lower, emphasizing the psychosocial burden of VPI, warranting psychosocial support in long-term care.

**Table 4:** Distribution of VELO scores among domain items.

VELO domain-item	Parent VELO	Youth VELO
	Mean ± SD	Mean ± SD
Speech limitation		
1. Air comes out my nose when I talk.	76.91 ± 30.77	76.74 ± 29.47
2. I run out of breath when I talk.	85.29 ± 24.72	85.96 ± 20.41
3. It is hard talking in long sentences.	70.29 ± 32.18	78.00 ± 25.98
4. My speech is too weak.	76.48 ± 29.72	78.04 ± 29.54
5. I have trouble being understood when I’m in a hurry.	60.88 ± 34.39	61.33 ± 34.70
6. My speech gets worse towards the end of the day.	89.85 ± 20.26	87.16 ± 22.38
7. My speech sounds different than other kids’.	65.98 ± 34.56	67.91 ± 33.50
Swallowing problems		
8. Liquids come out my nose while drinking.	81.47 ± 28.04	83.67 ± 23.06
9. Food comes out my nose while eating.	88.09 ± 23.43	95.00 ± 12.33
10. Others make fun of me when food or liquids come out my nose.	92.60 ± 19.21	93.92 ± 16.45
Situational difficulty		
11. My speech is hard for strangers to understand.	66.47 ± 31.49	68.67 ± 31.59
12. My speech is hard for friends to understand.	73.97 ± 29.21	82.67 ± 23.60
13. My speech is hard for family to understand.	83.24 ± 23.67	87.00 ± 20.29
14. I have trouble being understood when others can’t see my face, for example, in a car.	72.50 ± 31.68	80.33 ± 26.09
15. I have trouble being understood on the phone.	71.03 ± 30.45	76.35 ± 28.93
Emotional impact		
16. I am teased because of how I talk.	83.24 ± 26.89	82.67 ± 25.66
17. I get sad because of how I talk.	82.35 ± 29.50	83.00 ± 25.39
18. I get frustrated or give up when I am not understood.	80.59 ± 29.13	80.67 ± 25.53
19. I am shy because of how I talk.	78.68 ± 29.12	78.67 ± 28.08
Perception by others		
20. I am treated like I am not smart because of how I talk.	82.69 ± 27.68	87.50 ± 21.60
21. Others ignore me because of how I talk.	81.91 ± 27.74	83.67 ± 23.79
22. Others do not like to talk on the phone with me because of how I talk.	87.21 ± 23.90	92.00 ± 18.91
23. My family or friends tend to talk for me.	76.91 ± 27.60	79.67 ± 30.95
Caregiver impact		
24. I am worried or concerned about my child’s speech.	60.00 ± 37.53	NA
25. I find it difficult to understand my child.	83.68 ± 25.11	NA
26. My child’s speech problem slows me down or inconveniences me.	89.41 ± 23.83	NA

NA, not applicable.

### Predictors of inadequate QOL

The multivariate logistic regression model (Table 5) identified ONF and speech intelligibility deficits as strong independent predictors of inadequate QOL (*P* < .001). Hypernasality showed a borderline association but did not reach statistical significance in the adjusted model.

**Table 5:** Multivariate logistic regression analysis of significant factors influencing QOL of cleft palate patients.

Variable	*B*	SE	Wald	*P*	OR (95% CI)
Oronasal fistula	2.144	0.609	12.390	˂.001	8.531 (2.586-28.147)
Speech intelligibility deficient	2.181	0.609	12.817	˂.001	8.854 (2.683-29.222)
Hypernasality	–1.632	.846	3.717	.054	0.196 (0.037-1.027)

*B, β* coefficient; CI, confidence interval; OR, odds ratio; SE, standard error.

## Discussion

This study comprehensively evaluated the VPI-related QOL in cleft palate patients following primary palatoplasty using the VELO instrument and identified several critical clinical and functional predictors associated with poorer outcomes. The findings underscore the significant impact of speech outcomes, wound healing integrity, and age at repair on long-term QOL.

A notable finding was that patients with inadequate QOL were significantly older at the time of palatoplasty and evaluation, supporting the premise that delayed repair may compromise velopharyngeal function and subsequently affect speech development, social communication, and psychosocial adjustment. This reinforces the conclusions of prior studies, such as Jackson et al and the Sommerlad–Furlow series, which also found a strong correlation between later age at surgery and increased incidence of postoperative VPI and inferior speech outcomes.^1^^,^^9^^,^^25^, ^26^, ^27^, ^28^

The detrimental impact of late palatoplasty may be attributed to delayed establishment of normal velopharyngeal function during critical periods of speech acquisition. Moreover, delayed surgery may allow maladaptive compensatory articulation patterns to become engrained, leading to persistent speech deficits despite successful anatomical closure. As confirmed by receiver operating characteristic analysis in other studies, thresholds as early as 1.3/1.7 years have been associated with significantly improved speech outcomes.^1^^,^^28^ This suggests the necessity of timely intervention within developmental windows to optimize speech resonance and articulation.

Our results strongly support the centrality of speech-related outcomes, particularly speech intelligibility and velopharyngeal competence, in determining patient-perceived QOL. Patients with moderate to severe speech intelligibility deficits demonstrated disproportionately low VELO scores across all domains, including emotional and situational aspects. This aligns with prior findings that show speech intelligibility is one of the strongest predictors of both parental and youth-reported VELO scores.^14^^,^^18^^,^^20^

Consistent with previous studies validating the VELO instrument,^18^^,^^20^ our findings confirmed that speech-related parameters, including intelligibility, hypernasality, and nasal air emission, were significantly associated with total and domain-specific QOL scores. These results align with the well-established understanding that speech function plays an important role in patients’ daily experiences and psychosocial outcomes. While perceptual speech evaluation remains the clinical gold standard for assessing these features, QOL measures such as VELO may offer complementary insight by reflecting the patient’s lived experience and its broader impact on well-being.

In the multivariate analysis, ONF emerged as a significant independent predictor of lower QOL (odds ratio = 8.531), which aligns with previous reports identifying ONF as a complication that can negatively affect speech and swallowing.^1^ Symptoms related to ONF, such as nasal air escape, fluid leakage, and articulation challenges, likely contribute to social or emotional burdens captured by VELO scores. Prior studies, including those by Sakran et al and Parwaz et al, have highlighted factors such as cleft width and surgical tension that influence fistula risk.^1^^,^^9^^,^^29^, ^30^, ^31^ While principles such as adequate mobilization and tension-free closure are already standard in cleft surgery, our findings highlight the broader patient-reported impact of ONF and support ongoing efforts to minimize its occurrence.

In addition to fistula formation, speech intelligibility emerged as an equally strong independent predictor of reduced QOL, with patients demonstrating intelligibility deficits being nearly nine times more likely to experience inadequate QOL (odds ratio = 8.854; 95% confidence interval: 2.683-29.222). This underscores the central role of speech clarity in shaping both functional and psychosocial outcomes, further reinforcing that optimizing speech performance, through early repair, rigorous monitoring, and timely speech therapy, must remain a primary objective in cleft care.

The VELO questionnaire proved to be a robust, patient-centred tool capable of capturing the multidimensional burden of VPI. Notably, the emotional and social domains, especially items related to teasing, frustration, and being perceived as less intelligent, consistently scored lower in patients with VPI, highlighting the psychosocial impact of speech impairment. Youth-reported outcomes largely mirrored those of parents; however, some variation was noted, particularly in the swallowing domain, which did not differ significantly between VPI and non-VPI groups in the youth report. This discrepancy likely reflects reduced awareness or underreporting of swallowing difficulties among children compared with their caregivers, and reinforces the importance of incorporating both caregiver and patient perspectives in QOL evaluations. These results echo findings from prior validation studies that identified similar trends, particularly among parental proxies who tend to report more severe QOL impairments than the youth themselves.^4^^,^^19^

In the present study, concordance between parent and youth VELO scores was strong, supported by a high correlation and comparable total scores across informants. Only a small subset of cases (7.5%) demonstrated discordant classifications, typically involving youth scores close to the cutoff and not indicative of major perceptual differences. In such instances, we relied on the classification consistent with professional speech evaluation, the clinical gold standard, to ensure alignment between patient-reported outcomes and objective clinical assessment. These findings further underscore the complementary value of integrating both parent and youth perspectives, as each contributes unique insights into the child’s lived experience beyond what clinical examination alone can capture.

The findings of this study have several important clinical implications. First, early palatoplasty, ideally before 18 months of age, should be prioritized whenever feasible, as timely intervention is critical to minimizing long-term speech deficits and QOL impairments. In addition to early repair, postoperative care should extend beyond anatomical evaluation to focus on functional outcomes. This includes routine assessment of speech parameters and integration of VPI-related QOL measures, such as the VELO instrument, to capture the patient’s lived experience. Given the complex and multifactorial nature of VPI, comprehensive multidisciplinary management is essential. Collaboration among cleft surgeons, SLPs, psychologists, and social workers is necessary to address the wide-ranging effects of VPI on speech, emotional well-being, and social functioning. Finally, the VELO instrument should be incorporated into regular follow-up as a standardized PROM, especially in resource-limited or rural settings where access to specialized speech services may be restricted.

The broader influence of orofacial function on general health has been highlighted in recent literature. For example, Kim et al^32^ reported a significant association between masticatory difficulty and chronic cough in a large Korean population, underscoring how oral functional impairments may contribute to or exacerbate respiratory symptoms. Although the mechanisms differ from velopharyngeal dysfunction, their findings reinforce the concept that disturbances in oral and oropharyngeal function can have systemic repercussions beyond the immediate anatomical defect. In the context of cleft palate, our results similarly demonstrate that functional impairments, particularly those affecting speech, nasal airflow, and swallowing, extend beyond mechanical limitations to influence psychosocial well-being and overall QOL. Together, these studies highlight the importance of interdisciplinary care and the need to consider the wider health implications of orofacial dysfunction.

It should be noted that the youth version of the VELO questionnaire was completed only by patients aged 8 years and older at the time of evaluation/administration, in accordance with instrument guidelines. This does not introduce selection bias related to surgical age, as surgery occurred earlier with a wide age range (0.20-27 years), and the long follow-up period (1-15 years) allowed younger surgical candidates to reach the required age for youth self-report. Nonetheless, age-related developmental factors, such as greater social awareness, school experiences, and varying exposure to speech therapy, may influence youth perceptions of QOL independently of surgical outcome. These factors could contribute to subtle differences between parent and youth ratings and should be considered when interpreting VELO-derived QOL measures.

While the study presents robust data, it is limited by its cross-sectional design, which restricts causal inferences and the ability to track changes in QOL over time. In addition, youth self-reports may be affected by recall bias or underreporting, particularly in emotionally sensitive domains. Importantly, none of the patients received standardized postoperative speech therapy, which may have influenced VELO outcomes. Future research should focus on longitudinal studies to track QOL changes over time and evaluate the effectiveness of secondary interventions such as speech therapy or fistula repair, as well as other multidisciplinary management approaches.

## Conclusions

The findings of this study highlight the substantial influence of speech outcomes and postoperative complications on the VPI-related QOL in cleft palate patients. Timely primary palatoplasty, vigilant management of ONFe, and individualized speech therapy are essential to optimizing both functional recovery and patient-perceived well-being. The VELO instrument demonstrated strong value in capturing the multidimensional effects of VPI, including speech, emotional, and social domains. These results support its ongoing use as a reliable PROM in both clinical follow-up and research settings.

## Data availability

The data that support the findings of this study are available from the corresponding author upon reasonable request.

## Funding

This research did not receive any funding sources or financial support for the conduct of the research and/or preparation of the article.

## Declaration of competing interest

All authors declare that they have no conflict of interest.
